# A Darwin Core dataset of scorpions (Arachnida, Scorpiones) from the Royal Belgian Institute of Natural Sciences (RBINS) Collections

**DOI:** 10.3897/BDJ.14.e188187

**Published:** 2026-08-21

**Authors:** Fabiola Durante, Lorenzo Prendini, Roberto Pizzolotto, Gladys Wérenne, Léon Baert, Tobias Musschoot, Wouter Dekoninck

**Affiliations:** 1 Università della Calabria, Rende (CS), Italy Università della Calabria Rende (CS) Italy; 2 American Museum of Natural History, New York, United States of America American Museum of Natural History New York United States of America; 3 Gembloux Agro-Bio Tech, University of Liège, Gembloux, Belgium Gembloux Agro-Bio Tech, University of Liège Gembloux Belgium; 4 Royal Belgian Institute of Natural Sciences, Brussels, Belgium Royal Belgian Institute of Natural Sciences Brussels Belgium; 5 Belgian Biodiversity Platform, Brussels, Belgium Belgian Biodiversity Platform Brussels Belgium

**Keywords:** collections standardisation, Darwin Core, data curation, FAIR principles, museum, Scorpiones

## Abstract

**Background:**

This data paper details the publication of a dataset derived from the scorpion (Order Scorpiones C.L. Koch, 1850) collections preserved at the Royal Belgian Institute of Natural Sciences (RBINS), Brussels. The dataset includes all 3,652 specimens mostly identified to genus or species level during a recent re-evaluation. To maximise accessibility and interoperability, the entire dataset was fully standardised using the Darwin Core (DwC) standard and published as open data. The published dataset comprises a significant collection of records, encompassing eleven families, 56 genera and 117 species of scorpions. Geographically, the specimens originate from a wide range of locations, in 59 countries. The records within this dataset span a substantial chronological period, with collection dates ranging from 1872 to 2023. Most of the specimens were collected during expeditions led by researchers of RBINS.

**New information:**

The mobilisation and standardisation of this rich historical collection provide a valuable resource for global biodiversity informatics, supporting crucial research in taxonomy, ecology and biogeography of the order Scorpiones.

## Introduction

Natural history museums, exemplified by the Royal Belgian Institute of Natural Sciences (RBINS), Brussels and similar institutions globally, function as fundamental pillars supporting scientific discovery, education and cultural preservation (Suarez and Tsutsui 2004, Bakker et al. 2020). The preserved specimens housed within these collections are essential tools for understanding global biodiversity. They provide the critical historical evidence needed to analyse human impacts, track the effects of climate change and investigate other pressing environmental and socioeconomic challenges (Suarez and Tsutsui 2004, Meineke et al. 2019, Schmitt et al. 2019).

RBINS (and its collections) traces its roots back to 1846, shortly after Belgium gained independence in 1830. It was established as a natural history museum in Brussels, initially focusing on geology, palaeontology and zoology. Today, RBINS is one of Europe’s leading scientific institutions, managing the third-largest natural science collection in Europe, encompassing approximately 37 million specimens, which serve as a critical reference tool for global research. The Institute holds the European label of ‘major research infrastructure’ and actively contributes to the development of national and European environmental policies (Royal Belgian Institute of Natural Sciences 2018, Theeten et al. 2019). Its scientific prominence is underscored by multidisciplinary research in areas such as biodiversity, ecosystem monitoring and the history of life and climate (Royal Belgian Institute of Natural Sciences 2018, Theeten et al. 2019).

The primary goal of this article is twofold. First, we aim to provide a complete checklist of the identified specimens of the arachnid Order Scorpiones C.L. Koch, 1850, housed within the RBINS collection. Second and for the first time, this study aims to present a list of those specimens for which the taxonomic determination remains incomplete (such as only identified to family or genus level), hence offering a comprehensive and up-to-date overview of the entire RBINS scorpion collection.

Open and standardised data are fundamental for accelerating scientific discovery and maximising research impact. Standardisation, adhering to principles like FAIR (Findable, Accessible, Interoperable, Reusable) (Wilkinson et al. 2016), ensures interoperability, allowing datasets from diverse sources to be seamlessly combined, compared and reused by different systems globally (Wieczorek et al. 2012). Open access fosters transparency and is crucial for reproducibility, enabling the scientific community to validate findings and efficiently build upon prior work (Theeten et al. 2019). This approach ultimately prevents duplication of effort and promotes collaboration.

### Materials and methods

At present, RBINS has 3,652 scorpions amongst its biological specimens. The detailed determination of this collection was carried out in two main phases. A small portion of the collection was first examined in 2010 by L. Baert. The collection was then thoroughly re-examined in 2023 and 2025 by the author, Lorenzo Prendini, who made new identifications and revised previous ones. This revision resulted in the complete identification of the collection, although some specimens have not been identified to species level.

**Figure 1.**
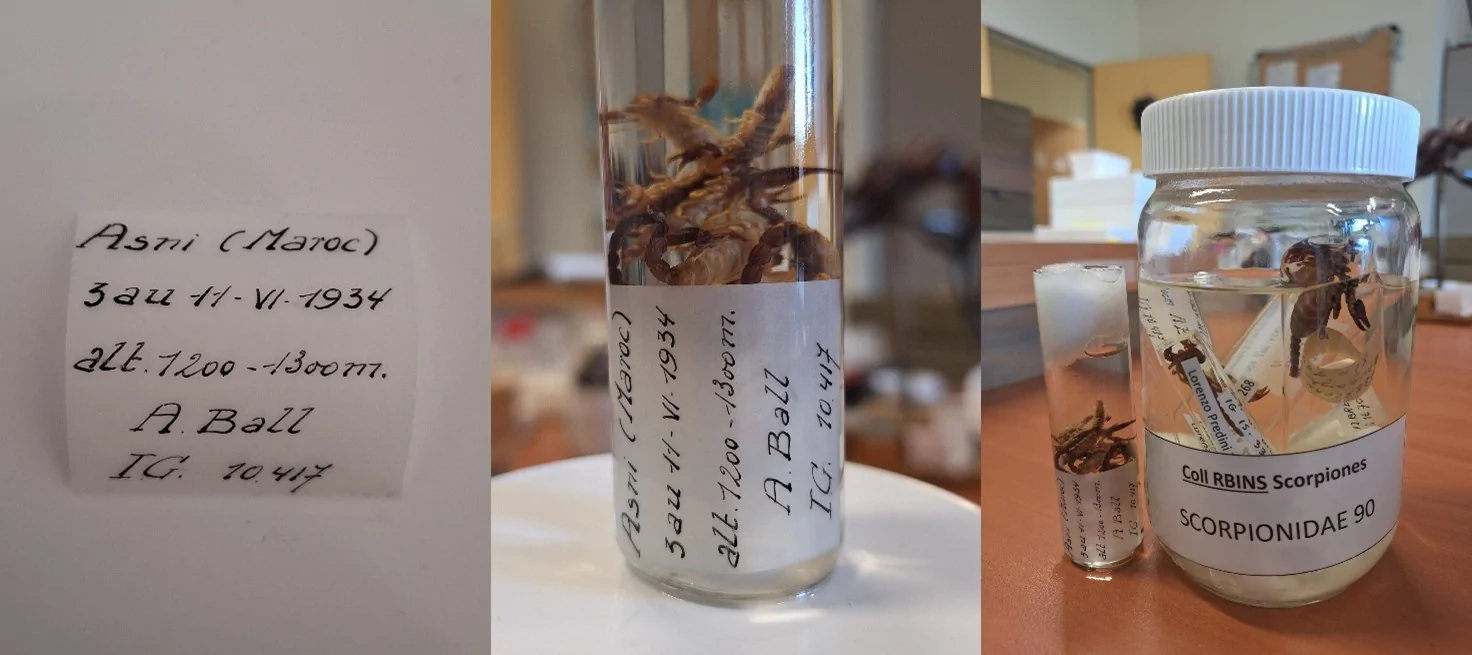
Example of a complete entomological label.

Following this work, the physical collection was entirely reorganised within the archives. It was decided to subdivide the identified specimens alphabetically, starting from the family and further down to genus level. Specifically, the collection is now stored in large jars labelled sequentially by family (e.g. Buthidae 1, Buthidae 2 etc.). Inside these larger containers, individual or multiple specimens (depending on number and size of the specimens) are housed in smaller vials, each accompanied by a detailed entomological label (Fig. 1).

The subsequent organisation within the archives varies, based on the identification status:

- Identified Specimens (to species level): The small vials containing these specimens are organised alphabetically by genus within the respective family jars. Each vial is accompanied by a complete label detailing the specimen’s full identification;
- Specimens identified above species level: For the portion of the collection that is not identified to the species level, the archiving order was established by following the family or sub-family to which it belongs and/or the exploratory campaign that led to its collection.

**Figure 2.**
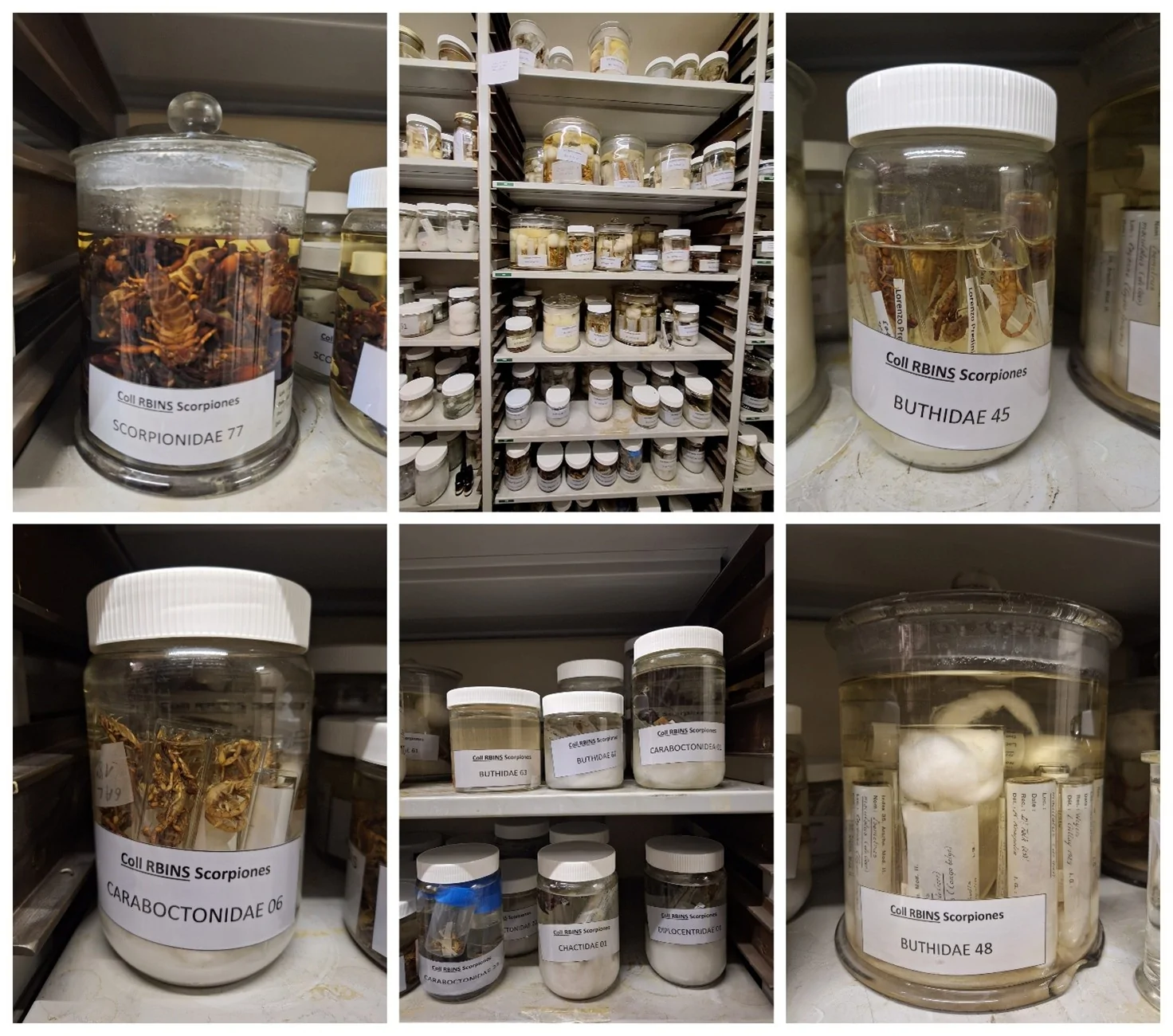
Organisation of RBINS scorpion collection with example jars and tubes.

Most of the scorpion specimens are preserved in 70% ethanol. However, a select portion of the collection, particularly more recently acquired material and specimens originating from the Galápagos Islands (Baert et al. 1995), is stored in 97% ethanol to ensure optimal DNA preservation, for example, for future molecular analyses. The specimens are directly stored in glass jars (Fig. 2). Some are stored in glass or plastic tubes in these jars. The collection contains very few microscopic slides and only a few dry mounted specimens. All specimens possess at least one label containing information on collection and identification.

Specimens are stored and sorted alphabetically by family and genus. This choice was motivated by its flexibility (Group et al. 2016). The numerous changes in scorpion phylogeny in recent years make a phylogenetic classification unstable. Each jar received a printed label with the name of the corresponding family and a chronological number. The old family labels and other labels were retained to ensure historical traceability. Given that, depending on its capacity, a jar may contain several species that are far apart in alphabetical order, the alphabetical system being a pragmatic curatorial choice that prioritises long-term physical stability over transient systematic accuracy (Boldgiv et al. 2025). In fluid collections, larger storage jars often act as secondary containers, holding numerous smaller vials (or tubes) with individual specimens. Under a strictly phylogenetic system, a major taxonomic revision (e.g. a family split or a genus transfer) would force the curator to physically re-label and relocate these heavy, fragile parent jars, risking damage and consuming significant time. By contrast, sorting alphabetically ensures that the jar's location remains fixed as long as the family or genus name starts with the same letter. Only the smaller, internal vials need re-labelling, reducing the risk and workload associated with reorganising the entire collection space. This method guarantees that a specimen's location is easily traceable even when scientific name changes (Wearn et al. 2013).

The taxonomy reported in the final checklist was primarily inserted and standardised using the classification system provided by the Global Biodiversity Information Facility (GBIF, https://www.gbif.org). Furthermore, to accurately assess species that have undergone recent nomenclatural or taxonomic changes, specific variations were evaluated by cross-referencing with the dataset available on Checklist Bank (https://www.checklistbank.org/).

The initial dataset was converted by standardising records using the widely adopted Darwin Core (DwC) biodiversity data standard (Wieczorek et al. 2012). Hence, a dataset was created that covered occurrences and then the various DwC terms were selected to translate all the information contained in the non-standardised version of the dataset.

All figures were created using R Statistical Software v. 4.4.2 (R Core Team 2024). The world map was obtained from Natural Earth (naturalearthdata.com) via R packages rnaturalearth v. 1.1.0 (Massicotte and South 2025) and rnaturalearthdata v. 1.0.0 (South et al. 2024). Data were processed with sf v. 1.0.20 (Pebesma 2018, Pebesma and Bivand 2023), tidyverse v. 2.0.0 (Wickham et al. 2019) and plotted with ggplot2 4.0.0 (Wickham 2016).

## Geographic coverage

### Description

Fifty-nine countries around the world.

## Taxonomic coverage

### Taxa included

**Table:** 

Rank	Scientific Name	Common Name
order	Scorpiones	Scorpions

## Temporal coverage

**Data range:** 1872-1-01 – 2023-9-01.

## Usage licence

### Usage licence

Other

### IP rights notes

CC BY-NC 4.0

## Data resources

### Data package title

RBINS Scorpions collection

### Resource link

https://doi.org/10.15468/t7ba89

### Number of data sets

1

### Data set 1.

#### Data set name

RBINS Scorpions collection (be_rbins_invertebrates_arachnida_scorpions)

#### Data format

Darwin Core table (tab delimited)

#### Character set

Occurrences

#### Description

Structure of the scorpions dataset, which consists of 1470 rows and 51 columns (Durante et al. 2026). The first column below gives the normative term names of the DwC terms used, the second column provides their definition (see https://dwc.tdwg.org/terms/for more details).

**Data set 1.** 

Column label	Column description
occurrenceID	Identifier for dwc:Occurrence (as opposed to particular digital record of dwc:Occurrence).
licence	Legal document giving official permission to use the resource.
basisOfRecord	Specific nature of data record.
individualCount	Number of individuals present at time of dwc:Occurrence.
lifeStage	Age class or life stage od dwc:Organism(s) at time dwc:Occurrence recorded.
sex	Sex of biological individual(s) represented in dwc:Occurrence.
occurrenceStatus	Statement about presence or absence of dwc:Taxon at dcterms:Location.
typeStatus	List (concatenated and separated) of nomenclatural types (type status, typified scientific name, publication) applied to subject.
scientificName	Full scientific name, with authorship and date information if known.
ScientificNameAuthorship	Authorship information for dwc:scientificName formatted according to conventions of applicable dwc:nomenclaturalCode.
nomenclaturalCode	Nomenclatural code (or codes in case of ambiregnal name) under which dwc:scientificName constructed.
kingdom	Full scientific name of kingdom in which dwc:Taxon classified.
phylum	Full scientific name of phylum or division in which dwc:Taxon classified.
class	Full scientific name of class in which dwc:Taxon classified.
order	Full scientific name of order in which dwc:Taxon classified.
family	Full scientific name of family in which dwc:Taxon classified.
genus	Full scientific name of genus in which dwc:Taxon classified.
parentNameUsage	Full name, with authorship and date information if known, of direct, most proximate higher-rank parent dwc:Taxon (in classification) of most specific element of dwc:scientificName.
specificEpithet	Name of first or species epithet of dwc:scientificName.
taxonRank	Taxonomic rank of most specific name in dwc:scientificName.
recordedBy	Person, group or organisation responsible for recording original dwc:Occurrence.
identifiedBy	List (concatenated and separated) of names of people, groups or organisations who assigned dwc:Taxon to subject.
dateIdentified	Date on which subject determined as representing dwc:Taxon.
identificationRemarks	Comments or notes about dwc:Identification.
identificationVerificationStatus	Categorical indicator of extent to which taxonomic identification verified to be correct.
type	Nature or genre of resource.
eventDate	Date-time or interval during which dwc:Event occurred. For occurrences, date-time when dwc:Event recorded. Not suitable for time in geological context.
habitat	Category or description of habitat in which dwc:Event occurred.
rightsHolder	Person or organisation owning or managing rights over resource.
institutionCode	Name (or acronym) in use by institution having custody of object(s) or information referred to in record.
institutionID	Identifier for institution having custody of object(s) or information referred to in record.
datasetID	Identifier for set of data. May be global unique identifier or identifier specific to collection or institution.
collectionCode	Name, acronym, coden or initialism identifying collection or dataset from which record derived.
ownerInstitutionCode	Name (or acronym) in use by institution having ownership of object(s) or information referred to in record.
country	Name of country or major administrative unit in which dcterms:Location occurs.
countryCode	Standard code for country in which dcterms:Location occurs.
stateProvince	Name of next smaller administrative region than country (state, province, canton, department, region etc.) in which dcterms:Location occurs.
locality	Specific description of place.
locationRemarks	Comments or notes about dcterms:Location.
verbatimElevation	Original description of elevation (altitude, usually above sea level) of dcterms:Location.
verbatimLatitude	Verbatim original latitude of dcterms:Location.
verbatimLongitude	Verbatim original longitude of dcterms:Location.
decimalLatitude	Geographical latitude (decimal degrees, using spatial reference system given in dwc:geodeticDatum) of geographical centre of dcterms:Location. Positive values north of Equator, negative values south thereof. Legal values lie between -90 and 90, inclusive.
decimalLongitude	Geographical longitude (decimal degrees, using spatial reference system given in dwc:geodeticDatum) of geographical centre of dcterms:Location. Positive values east of Greenwich Meridian, negative values west thereof. Legal values lie between -180 and 180, inclusive.
geodeticDatum	Ellipsoid, geodetic datum or spatial reference system (SRS) upon which geographical coordinates in dwc:decimalLatitude and dwc:decimalLongitude are based.
recordNumber	Identifier given to dwc:Occurrence at time recorded. Often serves as link between field notes and dwc:Occurrence record, such as specimen collector's number.
occurrenceRemarks	Comments or notes about dwc:Occurrence.
samplingProtocol	Names of, references to, or descriptions of methods or protocols used during dwc:Event.
materialEntityID	An identifier for a particular instance of a dwc:MaterialEntity.
otherCatalogNumbers	A list of previous or alternative fully qualified catalogue numbers or other human-used identifiers for the same dwc:Occurrence, whether in the current or any other dataset or collection.
infraspecificEpithet	The name of the lowest or terminal infraspecific epithet of the dwc:scientificName, excluding any rank designation.

## Additional information

**Results**

The dataset comprises eleven scorpion families: Bothriuridae Simon, 1880; Buthidae C.L. Koch, 1837; Caraboctonidae Kraepelin, 1905; Chactidae Pocock, 1893; Chaerilidae Pocock, 1893; Diplocentridae Karsch, 1880; Euscorpiidae Laurie, 1896; Hormuridae Laurie, 1896; Scorpionidae Latreille, 1802; Scorpiopidae Kraepelin, 1905; and Vaejovidae Thorell, 1876. The largest number of individuals in the collection (1,657) belong to Buthidae, followed by Scorpionidae (823) and Caraboctonidae (727).

Fifty-six genera and 117 species are represented in the collection. The genus represented by the most individuals is *Hadruroides* Pocock, 1893 with 720 individuals, followed by *Hottentotta* Birula, 1908 (641), *Pandipalpus* Rossi, 2015 (496), *Lychas* C.L. Koch, 1845 (289), *Isometrus* Ehrenberg, 1828 (145), *Scorpio* Linnaeus, 1758 (127) and *Babycurus* Karsch, 1886 (124).

**Figure 3.**
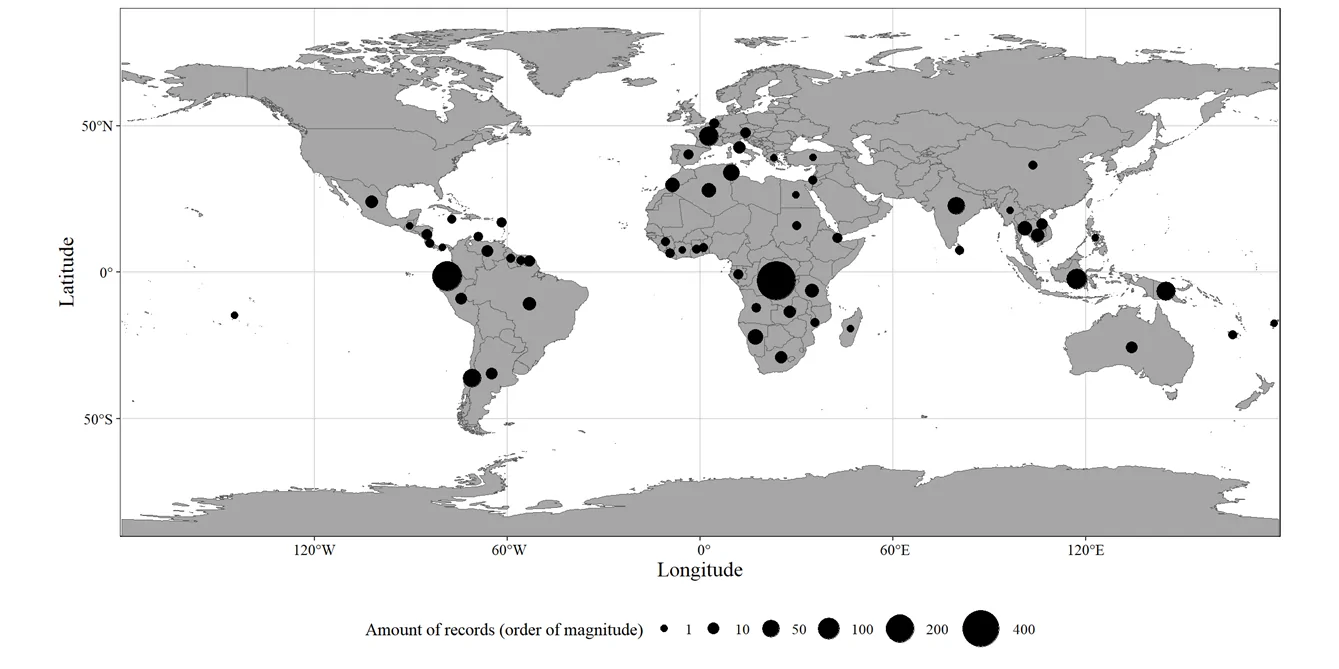
Origins of the records. Location of countries from which scorpions were collected. Area of circles is proportional to number of records. Values in legend provide reference points. Background map prepared with Natural Earth (https://www.naturalearthdata.com).

The dataset includes specimens from 59 countries (Table 1, Fig. 3). Amongst these, the countries with the largest number of individuals are the Democratic Republic of the Congo, with 1364 individuals and Ecuador (Galápagos Islands), with 729 individuals.

**Figure 4.**
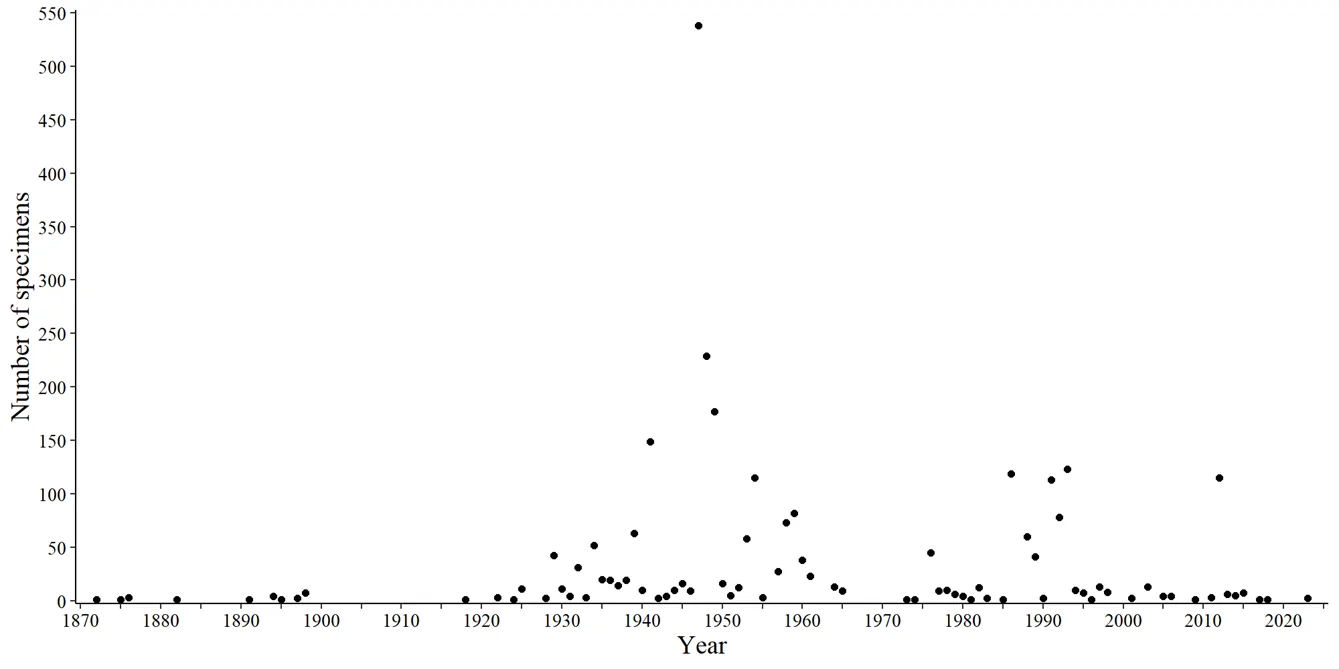
Annual number of specimens. Total number of scorpion specimens recorded each year between the first record in 1873 and the most recent in 2023.

In terms of the chronological distribution of the collections, the oldest specimen originates from Rio de Janeiro (Brazil) where it was collected in 1872, whereas the most recent was acquired in 2023 in Bruges (Belgium) (Fig. 4). The most frequent years are those between 1940 and 1950, which mostly represents sampling in the Upemba National Park by G.F. de Witte. There is no time reference for 380 records.

Gaston François de Witte (1897–1980), a Belgian naturalist and collector whose work greatly expanded the herpetological, entomological, zoological and botanical collections of Belgian institutions, is the most represented collector. In 1920, De Witte became attached to the Musée du Congo Belge. Later, in 1937, he became curator at the Royal Museum of Natural History in Brussels (later RBINS). De Witte carried out numerous scientific expeditions in the territory formerly known as the Belgian Congo (present-day Democratic Republic of the Congo), including fieldwork in areas such as Upemba National Park (De Witte 1966).

### Conclusions

The online publication of the RBINS scorpion data and this associated data paper, represent a significant advancement in the curation and accessibility of the RBINS scorpion collection. For the first time, most of this historically significant collection has undergone a comprehensive systematic revision (by Lorenzo Prendini in 2023 and 2025), verifying the identity of thousands of specimens and establishing a robust foundation for future research.

The core value of this work lies in the standardisation and mobilisation of this heritage. By fully adopting the DwC standard, we have successfully transformed disparate physical labels and institutional records into a FAIR digital dataset. This is essential, as the inherent instability of scorpion phylogeny can cause classification systems to rapidly become outdated; however, the standardised DwC format ensures that the underlying occurrence data remains permanently accessible and comparable with other collections globally, regardless of future taxonomic changes (Wieczorek et al. 2012, Marques et al. 2024). This open data approach maximises the scientific return on curatorial investment and actively supports global efforts in biodiversity informatics (Reyserhove et al. 2020, Crandall et al. 2023).

While clinical reports, such as Biezakala Mudiandambu et al. (2012), underscore the prevalence of scorpion envenomation in the Democratic Republic of the Congo, the lack of precise taxonomic identification remains a significant barrier to effective public health strategies. By providing a robust historical and taxonomic framework for medically significant genera like *Hottentotta* Birula, 1908 and *Lychas* C.L. Koch, 1845, these data help to bridge this gap, offering a reliable reference for future epidemiological research and the documentation of harmful species distribution.

Furthermore, whereas the mobilisation of the identified specimens is a major accomplishment, future work should focus on georeferencing the 73 records without location information and the six generic localities (e.g. Pyrenees, Sahara). This spatial enrichment is vital for enabling accurate ecological niche modelling, conservation assessments and biogeographical studies, thereby maximising the usability of this rich historical resource. In conclusion, the publication of the RBINS scorpion dataset is a testament to the enduring value of natural history collections. It not only solidifies the systematic knowledge of the identified collection, but provides a clear roadmap for the curatorial and taxonomic tasks that lie ahead, serving as a call to action for the specialised research community.

## Figures and Tables

**Table 1.** List of countries from where the specimens originate.

**Country**	**Specimens**
Democratic Republic of the Congo	1364
Ecuador	729
India	214
Tunisia	174
France	157
Indonesia	127
Papua New Guinea	114
Namibia	111
Chile	99
Morocco	43
Austria	29
Thailand	28
South Africa	27
Tanzania	26
Peru	26
Algeria	24
Cambodia	23
Argentina	23
Brazil	22
Mexico	21
Australia	19
Venezuela	17
Zambia	16
Italy	16
French Guiana	10
Vietnam	9
Nicaragua	8
Belgium	5
Togo	5
Antigua	5
Djibouti	5
China	5
Ghana	4
Liberia	4
Gabon	4
Spain	4
Guinea	3
Angola	3
Curacao	3
Fiji	3
Jamaica	2
Sri Lanka	2
Guyana	2
New Caledonia	2
Guatemala	2
Costa Rica	2
Sudan	2
Suriname	2
Egypt	2
Mozambique	2
Israel	2
Myanmar	1
Philippines	1
Turkey	1
Madagascar	1
Panama	1
Ivory Coast	1
Greece	1
French Polynesia	1
No locality	74
